# Bayesian Estimation of the ntPET Model in Single-Scan Competition PET Studies

**DOI:** 10.3389/fphys.2020.00498

**Published:** 2020-05-19

**Authors:** Zacharie Irace, Inés Mérida, Jérôme Redouté, Clara Fonteneau, Marie-Françoise Suaud-Chagny, Jérôme Brunelin, Benjamin Vidal, Luc Zimmer, Anthonin Reilhac, Nicolas Costes

**Affiliations:** ^1^CERMEP-Life Imaging, Lyon, France; ^2^SIEMENS Healthcare SAS, Saint Denis, France; ^3^INSERM U1028, CNRS UMR5292, Lyon Neuroscience Research Center, Psychiatric Disorders: from Resistance to Response Team, Lyon, France; ^4^Université Claude Bernard Lyon 1, Lyon, France; ^5^Centre Hospitalier Le Vinatier, Lyon, France; ^6^Hospices Civils de Lyon, Lyon, France; ^7^Clinical Imaging Research Centre, National University of Singapore, Singapore, Singapore

**Keywords:** brain imaging, PET, kinetic modeling, competition model, endogenous neurotransmitter release, lp-ntPET, Bayesian inference

## Abstract

This paper proposes an innovative method, named b-ntPET, for solving a competition model in PET. The model is built upon the state-of-the-art method called lp-ntPET. It consists in identifying the parameters of the PET kinetic model relative to a reference region that rule the steady state exchanges, together with the identification of four additional parameters defining a displacement curve caused by an endogenous neurotransmitter discharge, or by a competing injected drug targeting the same receptors as the PET tracer. The resolution process of lp-ntPET is however suboptimal due to the use of discretized basis functions, and is very sensitive to noise, limiting its sensitivity and accuracy. Contrary to the original method, our proposed resolution approach first estimates the probability distribution of the unknown parameters using Markov-Chain Monte-Carlo sampling, distributions from which the estimates are then inferred. In addition, and for increased robustness, the noise level is jointly estimated with the parameters of the model. Finally, the resolution is formulated in a Bayesian framework, allowing the introduction of prior knowledge on the parameters to guide the estimation process toward realistic solutions. The performance of our method was first assessed and compared head-to-head with the reference method lp-ntPET using well-controlled realistic simulated data. The results showed that the b-ntPET method is substantially more robust to noise and much more sensitive and accurate than lp-ntPET. We then applied the model to experimental animal data acquired in pharmacological challenge studies and human data with endogenous releases induced by transcranial direct current stimulation. In the drug challenge experiment on cats using [^18^F]MPPF, a serotoninergic 1A antagonist radioligand, b-ntPET measured a dose response associated with the amount of the challenged injected concurrent 5-HT1A agonist, where lp-ntPET failed. In human [^11^C]raclopride experiment, contrary to lp-ntPET, b-ntPET successfully detected significant endogenous dopamine releases induced by the stimulation. In conclusion, our results showed that the proposed method b-ntPET has similar performance to lp-ntPET for detecting displacements, but with higher resistance to noise and better robustness to various experimental contexts. These improvements lead to the possibility of detecting and characterizing dynamic drug occupancy from a single PET scan more efficiently.

## 1. Introduction

Positron emission tomography (PET) is a functional 3D *in vivo* imaging technique that allows to visualize and quantify with a very high sensitivity the local concentration of an injected radiotracer molecule. In neuroimaging, PET allows the investigation of key aspects of neurotransmission systems and provides important measurements such as the concentrations in presynaptic transporters and postsynaptic receptors in living human brains. PET data acquired dynamically are commonly analyzed using reference region models (Lammertsma and Hume, 1996; Lammertsma et al., 1996; Gunn et al., 1997) that are built on the assumption that the system under investigation is in steady state regime. In 1995, both Fisher et al. (1995) and Morris et al. (1995) advanced the possibility to use PET to detect dynamic changes in receptor binding or receptor occupancy occurring during activation studies. Their theory relied on the hypothesis that a cognitive task increases the firing rate of the involved neurons, leading to a release of endogenous neurotransmitter in the synaptic level with a measurable effect on the PET kinetics. This idea was then extended to using PET to reveal transient alterations caused by endogenous or exogenous competing binding compounds, as long as the PET tracer fulfills the pharmacokinetic characteristics set forth in Morris et al. (1995). However, the conventional reference region models are invalid in non-steady state conditions as they assume that the parameters to be estimated remain constant over the duration of the study. Consequently, one of the challenges for PET neuroimaging experiments became the design of robust and reliable kinetic analysis approaches with an integrated competition model to account for transient changes in kinetic binding and receptor occupancy in both low and high target density regions.

Several kinetic theories have been developed for non steady-state systems and related resolution methods have been designed to detect and characterize changes in ligand binding during a single PET scan. Alpert et al. (2003) proposed a linear extension of the reference region models, named LSSRM, that includes a time-varying efflux rate terms. The LSSRM model allows the statistical detection of a change in tracer binding, but it does not characterize the modulation. In fact, it assumes that competing endogenous releases or drug effects are instantaneous, maximal at time of stimulation and decay exponentially to baseline thereafter. Any violations of these assumptions might result in decreased sensitivity and specificity. Several kinetic models and associated resolution methods, collectively referred to as “ntPET” for neurotransmitter PET, with less stringent assumptions have then been proposed (Morris et al., 2005; Constantinescu et al., 2007; Normandin and Morris, 2008; Normandin et al., 2012). Among these methods, Normandin et al. (2012) proposed a linear parametric ntPET (lp-ntPET) as an extension of the LSSRM model that uses gamma variate functions spanning a wide range of feasible shapes, times of onset and duration to characterize the time course of the competing compound. While LSSRM uses three parameters to describe the neurotransmitter release (in addition to three parameters describing the transport of the tracer through the brain to blood barrier, and its binding at equilibrium), the lp-ntPET model uses four for the release characterization (seven in total). Normandin et al. proposed to handle the estimation of the non-linear parameters by discretizing them and employing a predefined library of basis functions. The other parameters are resolved using Weighted Least Squares (WLS) or Non-Negative Least Squares (NNLS) optimization.

Several issues are associated with this resolution approach. First, the use of basis functions leads to a poor accuracy of parameter estimation due to their discretization. In addition, the quality of fit and accuracy of solutions greatly depends on the choice of the basis function (Liu and Morris, 2019). This dependency can result in a moderate sensitivity and an uncertain specificity for the detection of the transient change, and a loss of accuracy for its characterization. Moreover, the lp-ntPET model is over-determined, i.e., different sets of parameters may produce similar response curves. In such context, the least squares-based approaches that are used in the lp-ntPET method to estimate the linear parameters may lead to a lack of reproducibility. Finally, the least squares method is known to be highly sensitive to noise and the classic approach may be unreliable in real applications where high levels of noise are not unusual, especially in small Regions Of Interest (ROIs). An alternative method that does not rely on basis functions is described in Fan et al. (2016), who proposed an estimation method based on Approximate Bayesian Computing (ABC). Their investigations on simplistic simulated data are encouraging but did not lead to conclusive results for real studies.

In this work, we introduce a novel resolution method for ntPET models, called b-ntPET, whereby the parameter estimation relies on a Markov-Chain Monte-Carlo (MCMC) sampling in a Bayesian framework. The presented methodology assesses the probability distribution of the unknown parameters, and consequently allows the quantification of the uncertainty of the parameter estimates. Moreover, we hypothesized that the integration of a priori information on the model parameters, as allowed in this Bayesian framework, will tackle the identifiability problem by reducing the set of eligible solutions. Finally, we proposed to jointly estimate the noise level with the parameters of the model, for increased robustness. We validated our b-ntPET method and compared its performance against the reference lp-ntPET approach using realistic simulated datasets as well as preclinical and clinical data.

## 2. Methods

### 2.1. Modeling Tracer Competition

In the Simplified Reference Tissue Model (SRTM) (Lammertsma et al., 1996), the kinetic *C*_*T*_(*t*) of a target region is defined relative to the kinetic *C*_*R*_(*t*) of a reference region.

(1) $$\begin{array}{r} C_{T}\left(t\right)=R_{1}C_{R}\left(t\right)+k_{2}\int_{0}^{t}C_{R}\left(u\right)du-k_{2a}\int_{0}^{t}C_{T}\left(u\right)du \end{array}$$

**Figure 1:**
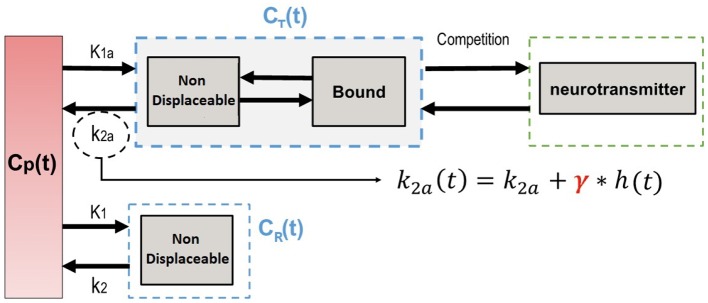
Compartment model illustrating a competition model.

Where *R*_1_ = *K*_1*a*_/*K*_1_ is the local rate of delivery in the target tissue relative to the reference tissue, *k*_2_ is the transfer rate constant from tissue to blood in the reference region, and *k*2*a* is the transfer rate constant from tissue to blood in the target region. Alpert et al. (2003) generalized this model by considering a time-varying efflux rate *k*_2*a*_(*t*) that reflects the competition between the radioligand and the endogenous neurotransmitter at the receptor sites:

(2) $$\begin{array}{r} k_{2a}\left(t\right)=k_{2a}+\gamma \cdot h\left(t\right) \end{array}$$

where γ represents the magnitude of transient effects and the function *h*(*t*) characterizes the endogenous neurotransmitter discharge or an exogenous concurrent drug concentration level (Figure 1).

This leads to the following operational equation to model the time-activity curve (TAC) *C*_*T*_(*t*) of a tissue of interest:

(3) $$\begin{array}{l} C_{T}\left(t\right)=R_{1}C_{R}\left(t\right)+k_{2}\int_{0}^{t}C_{R}\left(u\right)du-k_{2a}\int_{0}^{t}C_{T}\left(u\right)du-\gamma \\ \;\int_{0}^{t}C_{t}\left(u\right)h\left(u\right)du \end{array}$$

With regard to the choice of *h*(*t*), the exponential function initially proposed in LSSRM (Alpert et al., 2003) has been extended by Normandin et al. (2012) to:

(4) $$\begin{array}{l} h\left(t\right)=\{\begin{array}{ll} {\left(\frac{t-t_{D}}{t_{P}-t_{D}}\right)}^{\alpha }\exp\left(\alpha \left[1-\frac{t-t_{D}}{t_{P}-t_{D}}\right]\right), & \forall t\geq t_{D}.\\ 0, & \forall t\leq t_{D}. \end{array} \end{array}$$

This model is driven by seven parameters among which four allow to fully characterize the discharge by expressing its magnitude (γ), the time at which it begins (*t*_*D*_), the time at its peak (*t*_*P*_) and its global sharpness (α). The combination of these quadruplets results in a set of possible response functions. It is worth noting that the model is over-determined and various combinations of these parameters can result in producing similar shapes of the release. This results in an identifiability issue that may disturb the robustness and the reproducibility of the estimation methods, especially in the presence of high noise.

In the original method from Normandin et al. (2012), the linear coefficients (*R*_1_, *k*_2_, *k*_2*a*_, γ) were estimated with a weighted least-squares method. Since the other parameters (*t*_*D*_, *t*_*P*_, α) are non-linear, optimal (*R*_1_, *k*_2_, *k*_2*a*_, γ)_*i*_ were estimated for each *h*_*i*_(*t*) from a set of basis functions driven by the parameters (*t*_*D*_, *t*_*P*_, α)_*i*_. The combination of these three parameters that lead to the best fitting of the measurements determined the best *h*_*i*_(*t*) and its associated (*R*_1_, *k*_2_, *k*_2*a*_, γ)_*i*_ parameters.

### 2.2. Overview of the Proposed Estimation Method

**Figure 2:**
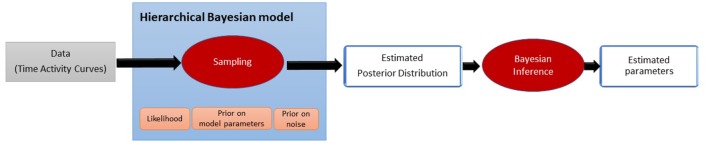
Pipeline illustrating the proposed estimation process. The estimation of the posterior distribution is an intermediate step before estimating the final value of the parameters.

The method aims at estimating the parameters driving the tracer displacement model defined by Equations (3) and (4). In the Bayesian paradigm, the parameters to be estimated are no longer considered fixed quantities but random variables on which prior knowledge can be applied. The proposed approach estimates the probability distribution of the parameters given the measurements, also called posterior probability, as an intermediate step. This new paradigm brings more flexibility toward noise and allows to quantify the uncertainty of the estimations. Figure 2 sums up the whole estimation process.

First, the posterior has to be defined by setting a hierarchical Bayesian model. The Bayesian model takes into account the model of the noise (likelihood) and the amount of prior information we may have on the unknown parameters. Due to the complexity of the posterior distribution, its explicit expression is unknown. In this work, we propose to estimate the posterior probability using a sampling technique (Robert and Casella, 1999). This approach draws a relevant amount of samples that are asymptotically distributed according to the posterior distribution. From these estimated probability distributions, Bayesian inference is made to determine the final value of each parameter. The steps of the method are described in the subsections below.

### 2.3. Hierarchical Bayesian Model

After reparameterization Δ_*t*_ = *t*_*P*_ − *t*_*D*_ for convenience, let us define **Θ** = {_θ_*k*_}*k* = 1…7_ = (*R*_1_, *k*_2_, *k*_2*a*_, γ, *t*_*D*_, Δ_*t*_, α) the set of unknown parameters and ***Y*** = (*y*_1_, ⋯ , *y*_*N*_) the measured Time Activity Curve (TAC), where *y*_*n*_ is the measured activity at frame *n*. In that study, the variance of the noise ω^2^ is also considered unknown and will be jointly estimated with the model parameters **Θ**. We want to estimate the joint probability *p*(**Θ**, ω^2^|***Y***) of the parameters **Θ** and the noise level ω^2^ given the measurements ***Y***. The Bayes rule states that:

(5) $$\begin{array}{r} p\left(\Theta ,\omega^{2}|Y\right)\propto p\left(Y|\Theta ,\omega^{2}\right)\cdot \pi \left(\Theta \right)\cdot \pi \left(\omega^{2}\right) \end{array}$$

Where *p*(**Θ**, ω^2^|***Y***) is called the *posterior* distribution, ∝ means *proportional to*, *p*(***Y***|**Θ**, ω^2^) is called the *likelihood* and corresponds to the noise model, π(**Θ**) is the *prior* that reflects the knowledge we may have on the model parameters and π(ω^2^) is the *prior* on the variance of the noise. The likelihood and both priors on the model parameters and on the noise level are defined in the subsections below.

#### 2.3.1. Likelihood

We assume that the noise at each frame is independent from the noise of the other frames. The likelihood can then be factorized as:

(6) $$\begin{array}{r} p\left(Y|\Theta ,\omega^{2}\right)=\overset{N}{\underset{n=1}{\prod }}p\left(y_{n}|\Theta ,\omega^{2}\right) \end{array}$$

with *y*_*n*_ the measured value at frame *n* of the dynamic PET image. The likelihood at each frame $p\left(y_{n}|\Theta ,\omega^{2}\right)$ is considered normally distributed around the value expected by the model at the middle of the frame **Θ**(*n*) and with the variance $\omega_{n}^{2}$:

(7) $$\begin{array}{r} y_{n}|\Theta ,\omega^{2}\sim N\left(\Theta \left(n\right),\omega_{n}^{2}\right) \end{array}$$

The variance $\omega_{n}^{2}$ corresponds to the noise level of each frame. The noise levels vary from one frame to another, but can be linked considering the popular weighting factors used to model the noise, such as the activity **Θ**(*n*) itself, the decay factor *t*_*n*_ and the frame duration *d*_*n*_ according to the following formula:

(8) $$\begin{array}{r} \omega_{n}^{2}=\omega^{2}\frac{\Theta \left(n\right)}{t_{n}\cdot d_{n}}. \end{array}$$

The value ω^2^ corresponds to a noise level that is representative of the whole TAC and is estimated by the algorithm jointly with the parameters of the model.

#### 2.3.2. Prior Definition

##### 2.3.2.1. Prior on the Model Parameters

Assuming that the model parameters are independent, the joint prior distribution of the vector **Θ** is:

(9) $$\begin{array}{r} \pi \left(\Theta \right)=\overset{7}{\underset{k=1}{\prod }}\pi \left(\theta_{k}\right)=\pi \left(R_{1}\right)\cdot \pi \left(k_{2}\right)\cdot \pi \left(k_{2a}\right)\cdot \pi \left(\gamma \right)\cdot \pi \left(t_{D}\right)\cdot \pi \left(\Delta_{t}\right)\cdot \pi \left(\alpha \right) \end{array}$$

The choice of prior distributions can be based on prior information obtained from preliminary studies or on other known information from the protocol design. The more realistic the prior distributions, the more accurate the solution. While more attention should be paid to the design of the priors for optimal results, in this work, we deliberately chose non-informative priors on the model parameters, so the quality of the results can be credited to the resolution approach only and not to overly helping prior information. To this end, we used a uniform prior distribution π(θ_*k*_) for each parameter, with intervals largely covering its plausible value range:

(10) $$\begin{array}{r} \theta_{k}\sim U\left(\theta_{k}^{\min},\theta_{k}^{\max}\right) \end{array}$$

##### 2.3.2.2. Prior on the noise variance

The prior on the noise variance π(ω^2^) is chosen as an inverse-gamma distribution with hyper-parameters *a*_0_ and *b*_0_.

(11) $$\begin{array}{r} \omega^{2}\sim \Gamma^{-1}\left(a_{0},b_{0}\right) \end{array}$$

This choice is motivated by the fact that the inverse-gamma distribution is a conjugate prior of the variance for the normal distribution (see next section). The inverse-gamma distribution also ensures that ω^2^ is positive.

The values of *a*_0_ and *b*_0_ depict the a priori information we may have on the variance of the noise. In our case, they are chosen so that π(ω^2^) is centered on an empirical estimation of the variance of the TAC and the variance (the variance of ω^2^ itself) is set to a very large value so that prior assumption on ω^2^ are non-informative.

### 2.4. Sampling

The posterior distribution defined in (5) is too complex to be expressed in closed-form and deriving explicit solutions is intractable. We propose to estimate the posterior distribution using Monte-Carlo sampling in the parameter space. The idea is to draw a sufficient amount of samples that are asymptotically distributed according to the posterior distribution, also called target distribution. More precisely, a hybrid Metropolis-within-Gibbs sampler has been implemented, where the step size of each chain is adjusted to ensure an optimal mixing behavior.

#### 2.4.1. Gibbs

The target distribution *p*(**Θ**, ω^2^|***Y***) is defined in an 8-dimensional space. Sampling the whole random vector **Θ** directly is challenging because of the anisotropic nature of the parameter space (the individual parameters behave in very different ways). The Gibbs sampler considers a random vector as a set of individual random variables. It allows to draw samples of each variable separately according to their univariate posterior conditional distribution $p\left(\theta_{k}|Y,\omega^{2},\Theta_{-k}\right)$ for each θ_*k*_, where **Θ**_−*k*_ is **Θ** without θ_*k*_, and *p*(ω^2^|***Y***, **Θ**) for ω^2^. By sampling iteratively each parameter at a time according to its associated posterior conditional distribution, the Gibbs algorithm defines a Markov Chain whose stationary distribution is the target distribution *p*(**Θ**, ω^2^|***Y***). The parameters θ_*k*_ are sampled according to their posterior conditional using a Metropolis-Hastings process (see next section), leading to a so-called Metropolis-within-Gibbs algorithm (see Robert and Casella, 1999 for more details).

#### 2.4.2. Metropolis-Hastings for the Model Parameters

The Random Walk Metropolis-Hastings (RWMH) is one of the most common MCMC algorithms. The idea is to draw a sequence of random samples, where the value of each sample is obtained relatively to the value of the previous one. More precisely, given the value of the jth sample $\theta_{k}^{j}$, a new sample $\theta_{k}^{*}$ is proposed according to the following law of motion: $\theta_{k}^{*}=\theta_{k}^{j}+\epsilon_{k}\cdot w^{j}$, where *w*^*j*^ denotes a Brownian motion and ϵ_*k*_ is a scaling factor. In order that the sequence of samples are distributed according to the target density, the proposed sample $\theta_{k}^{*}$ is accepted or rejected with probability $\min\left(1,\frac{p\left(\theta_{k}^{*}|Y,\Theta_{-k},\omega^{2}\right)}{p\left(\theta_{k}^{j}|Y,\Theta_{-k},\omega^{2}\right)}\right)$. If the proposed sample $\theta_{k}^{*}$ is accepted, the value of the new sample is set to $\theta_{k}^{j+1}=\theta_{k}^{*}$, if it is rejected, the value of the new sample stays the same $\theta_{k}^{j+1}=\theta_{k}^{j}$. An efficient mixing of the target distribution requires that the acceptance rate is close to $\frac{1}{2}$. The step size ϵ_*k*_ must be chosen accordingly.

The posterior conditional distribution for each model parameter θ_*k*_ is $p\left(\theta_{k}|Y,\Theta_{-k},\omega^{2}\right)\propto p\left(Y|\Theta ,\omega^{2}\right)\pi \left(\theta_{k}\right)$ where one recognizes the likelihood and the prior on θ_*k*_ defined in (6) and (10), respectively.

#### 2.4.3. Direct Sampling for the Noise Variance

The posterior conditional associated to the variance is *p*(ω^2^|***Y***, **Θ**) ∝ *p*(***Y***|**Θ**, ω^2^)π(ω^2^). Here, we take advantage of the fact that the noise is assumed to be normally distributed and that the inverse-gamma distribution is a conjugate prior of the variance for the normal distribution. More precisely, by choosing π(ω^2^) as an inverse-gamma distribution with hyper parameters *a*_0_ and *b*_0_, we know that *p*(ω^2^|***Y***, **Θ**) is also an inverse-gamma distribution with parameters $a=a_{0}+\frac{N}{2}$ and $b=b_{0}+\frac{1}{2}\sum_{n=1}^{N}\left(y_{n}-\Theta \left(n\right)\right)$. With a closed-form expression for the conditional posterior, it is then possible to draw samples for ω^2^ using direct sampling instead of an acceptance-rejection scheme. Note that ω^2^ is sampled and not ω.

#### 2.4.4. Step Size Calibration

To avoid difficulties due to anisotropy of the parameter space, each parameter has an adapted step size ϵ_*k*_. The choice of the step size ϵ_*k*_ has a direct impact on the efficiency of the sampling process. A large ϵ leads to a high rejection rate, and a low value provides highly correlated samples. In both cases, the algorithm would be characterized by excessively slow mixing. In the proposed method, the optimal values of ϵ_*k*_ are estimated empirically during a first phase called burn-in so the acceptance rate of the proposed samples reaches $\frac{1}{2}$. Samples drawn during the burn-in period are then withdrawn.

### 2.5. Bayesian Inference

The estimated posterior distribution is a result in itself, it can be exploited to perform model selection, to find if one or several modes pop up from the whole distribution, and allows to evaluate the degree of trust we may attribute to a detection. When it comes to extract the final value of the parameters from this distribution, one must perform inference and look for a suitable estimator.

Popular estimators are the Minimum Mean Square Error (MMSE) and the Maximum a Posteriori (MAP). MMSE corresponds to the expectancy of the distribution (or the mean of the samples). It is very robust but may be inappropriate if the posterior distribution is asymmetrical, has several modes, or when the model suffers from identifiability issues. MAP corresponds to the drawn sample whose posterior probability is the highest. However, the MAP estimator lacks accuracy and reproducibility when the MCMC samples are sparse in the parameter space.

In this work, we propose to resort to a mode-seeking algorithm to find the main mode of the target distribution. More precisely, we are looking for the Highest Posterior Density (HPD) region. HPD consists in localizing the smallest region containing a given percentage of the drawn samples. Once the interval has been found, we perform MMSE on the subset of the samples in that region to get the optimal values for each parameter. Since computing HPD on the multivariate distribution is very time consuming, and may be altered by the sparsity of the samples in a 8D space, we perform univariate HPD on the marginal distributions of each parameter. In this work, we looked for the smallest interval containing the arbitrary amount of 10% of the samples.

## 3. Material

The performance of the presented b-ntPET method was evaluated with three brain PET studies and compared to the reference lp-ntPET method. The first study consisted of 21 simulated realistic dynamic [^11^C]raclopride PET scans. This dataset was used to evaluate the accuracy of the method, as well as its robustness against noise in well-controlled conditions.

The second study consisted of experimental dynamic [^18^F]MPPF scans in cats involving a drug challenge with an agonist of the 1A sub-type serotonin (5-HT_1A_) receptor. This dataset was used to assess the quantification capacity of the method.

The third study consisted of experimental human dynamic [^11^C]raclopride acquisitions with a bolus-infusion protocol during which a transcranial direct current stimulation (tDCS) was applied. This third dataset was used to test the sensitivity of the method for detecting and characterizing the dopaminergic discharge induced by tDCS.

The model parameters were estimated with the proposed method b-ntPET, and compared with the lp-ntPET resolution. For b-ntPET, 55,000 samples were drawn including 5,000 samples of burn-in, in the three datasets.

### 3.1. Simulated [^11^C]raclopride PET Data and Ground Truth Determination

A total of 21 realistic dynamic brain PET scans corresponding to a 100-min bolus-infusion [^11^C]raclopride PET protocol were generated using the PET-SORTEO platform (Reilhac et al., 2004, 2005) simulating the performance of the Siemens Biograph mMR scanner (Reilhac et al., 2016). Each PET scan was simulated from a structurally different numerical brain model to account for inter-individual anatomical variability as well as using ideal TACs describing the time course of the tracer, including well controlled variations caused by dopaminergic discharges. Each brain model consisted of (1) a 3D attenuation numerical phantom that described the attenuation coefficients in order to account for photoelectric absorption, elastic (Rayleigh), and inelastic (Compton) scatterings of the photons in the human tissues during the simulation process, and (2) a 3D emission numerical phantom showing the emitting brain structures. Attenuation and emission phantoms were respectively constructed from the 3-tissue class binary image derived from a CT scan and from the parcellation of a T1 MRIs, both acquired on the same subject (Mérida, 2017).

Ideal TACs were defined for the simulation of each emitting brain structure using a full kinetic model involving the definition of a plasmatic input function (IF), from which tissue responses were generated. The ideal IF was defined by fitting experimentally measured IF data with a three exponential model:

$$\begin{array}{l} C_{p}\left(t\right)=\overset{3}{\underset{i=1}{\sum }}A_{i}\exp\left(-\left(t-t_{\text{peak}}\right)\cdot \frac{\log\left(2\right)}{T_{i}}\right) \end{array}$$

with (*T*_1_, *T*_2_, *T*_3_) = (4.28, 735.5, 183.5) sec, (*A*_1_, *A*_2_, *A*_3_) = (288.6, 1.1, 409.7) Bq/ml and *t*_peak_ = 110 sec. From this ideal plasmatic IF, TAC for the reference region was generated using a one-tissue compartment model $\frac{dC_{\text{ref}\left(t\right)}}{dt}=K_{1}^{\text{ref}}\cdot C_{p}\left(t\right)-k_{2}^{\text{ref}}\cdot C_{\text{ref}}\left(t\right)$ with $K_{1}^{\text{ref}}=0.0918$ mL/(min.g), $k_{2}^{\text{ref}}=0.242$ min^−1^ (Pappata et al., 2002; Alpert et al., 2003) and a calibration factor of 10.

**Figure 3:**
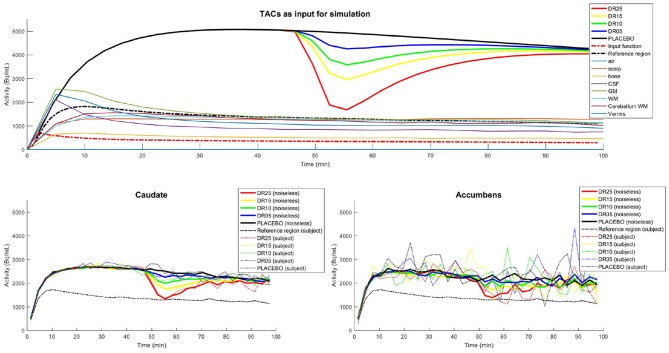
**(Top)** Ideal TACs used as input for the PET-SORTEO simulations. The three regions caudate, putamen, and accumbens nuclei share the same TACs. TACs corresponding to endogenous releases of dopamine of 0, 5, 10, 15, and 25%, (PLACEBO, DR05, DR10, DR15, and DR25) were used successively for the simulation of the left caudate, putamen, and accumbens nucleus. **(Bottom)** Caudate (left) and Accumbens (right) TACs measured from the images reconstructed from the simulated data: Mean of the measured TACs (noiseless TACs) over all subjects (solid line) and measured TACs from a single subject (dashed line). The TAC of the reference region is shown for the subject in the PLACEBO condition. Note that the magnitude of the TACs are not completely retrieved in the reconstructed images due to signal degradation caused by the limited spatial resolution of the system (partial volume effects). The accumbens is a smaller region than the caudate, which explains the higher level of noise in the TACs.

Ideal TACs that included modulations caused by endogenous dopamine release were then generated for caudate, putamen and accumbens using Equations (3) and (4), with *R*_1_ = 1.1540, *k*_2_ = 0.242, *k*_2*a*_ = 0.0653, *t*_*D*_ = 42min, *t*_*P*_ = 51min, α = 15 and five different magnitudes of dopamine release: γ = [0;0.035;0.078;0.1284;0.3] corresponding to displacement ratios (Mérida et al., 2018) of 0, 5, 10, 15, and 25 (hereafter referred to by placebo, DR05, DR10, DR15, and DR25 respectively). Endogenous release was lateralized to the left side of the brain and applied to the three structures of the striatum: caudate nucleus, nucleus accumbens, and putamen. Same input kinetics were used for all 21 subjects. TACs for the simulation of activity uptakes in surrounding cerebral regions of lesser interest as well as in extra cerebral regions (air, soft tissue, bone, CSF and ventricles, GM, WM, cerebellar WM, cerebellar vermis) were built from experimental PET/CT data measurements in lieu of from analytical calculation (Mérida, 2017). The whole set of TACs used in this simulated study is shown in Figure 3.

Simulated raw emission data of each subject was then rebinned and reconstructed into 33 time-frames of 3 min each with the OP-OSEM3D algorithm incorporating the point spread function (PSF) modeling, normalization as well as attenuation and scatter correction, and using 12 iterations of 21 subsets. A zoom of three was applied to the reconstructions, yielding a voxel size of 0.9 × 0.9 × 2.03 mm^3^ in a matrix of 256 × 256 × 127 voxels. TACs were finally extracted from simulated PET data for striatal ROIs and reference region using the emission phantom for brain structure parcellation.

Due to the degradation occurring during reconstruction (partial volume effects mainly), the activity levels measured from the reconstructed image are not systematically retrieved for small brain structures. These alterations have a direct impact on the kinetic parameters to be estimated. To propose a more realistic reference than the unachievable values set as input for simulations, we considered the mean of the measured TACs over all subjects, thereafter called *noiseless* TACs, for each condition. The reference value for *R*_1_, *k*_2_, and *k*_2*a*_ are set by fitting the noiseless TAC of the placebo condition with the SRTM model (Lammertsma and Hume, 1996). The reference values for the release parameters (γ, *t*_*D*_, Δ_*T*_, α) have then been defined by fitting the noiseless TACs of each condition with both lp-ntPET and b-ntPET methods.

The prior intervals for the b-ntPET method were set to $R_{1}\sim U\left(1,2\right)$, $k_{2}\sim U\left(0,0.5\right)$, $k_{2a}\sim U\left(0,0.1\right)$, γ~U(-0.5,0.5), $t_{D}\sim U\left(40,60\right)$, $\Delta_{t}\sim U\left(5,25\right)$, α~U(10,20). For the lp-ntPET approach, the basis functions have been chosen by setting the extreme values for *t*_*D*_ and α accordingly, with a step of 30 sec for *t*_*D*_ and *t*_*P*_, and a step of 0.5 for α.

### 3.2. Experimental [^18^F]MPPF Cat Brain PET Data

Four male cats underwent 90 min PET-MRI scans following a bolus injection-infusion of [^18^F]MPPF, a 5-HT_1A_ antagonist radiotracer, on an integrated Siemens Biograph mMR scanner. Each cat underwent four separate PET-MRI acquisitions: three involving a pharmacological challenge at 50 min with NLX-112, a 5-HT_1A_ agonist, injected at 0.04, 0.08, or 0.16 mg/kg, and one involving saline injection for control. Dynamic PET images were reconstructed from the acquired list-mode with 3D OP-OSEM algorithm, using point spread function modeling, normalization, scatter, and attenuation correction as well as with a zoom 4, yielding to a matrix of 256 × 256 × 127 pixels, with voxels of 0.7 × 0.7 × 2.03 mm^3^. PET images were realigned and registered into the same space using a multi-subject MRI template, coregistered with a labeled atlas defining standard regions of interest (Lancelot et al., 2010). Using this atlas, TACs for the hippocampus and cerebellum were extracted and modeled with the classic lp-ntPET method, and with the b-ntPET method to quantify and characterize the endogenous release. For additional information, the original study can be found in Vidal et al. (2018).

The prior intervals for the b-ntPET method have been set to $R_{1}\sim U\left(1,2\right)$, $k_{2}\sim U\left(0,0.5\right)$, $k_{2a}\sim U\left(0,0.1\right)$, γ~U(-0.5,0.5), $t_{D}\sim U\left(45,70\right)$, $\Delta_{t}\sim U\left(5,25\right)$, α~U(0,20). For the classic lp-ntPET approach, the basis functions were chosen by setting the extreme values for *t*_*D*_ and α accordingly, with a step of 30 s for *t*_*D*_ and *t*_*P*_, and a step of 0.5 for α.

### 3.3. Experimental [^11^C]raclopride Human Brain PET Data

Thirty-two healthy subjects (mean age = 25.25±3.55 years) underwent a 100 min PET acquisition on the Siemens PET/CT Biograph after the intravenous injection of [^11^C]raclopride (18 MBq + 2.6 MBq/kg) and followed by a constant infusion. During the collection of the PET data, each subject received a single 10 min tDCS session with intensity 2 mA, that started 40 min after the injection of the tracer. Subjects were divided in two parallel groups, active (*n* = 14) vs. sham (*n* = 18) bifrontal tDCS. A total of 20 successive frames of 5 min each was reconstructed with 3D OP-OSEM iterative algorithm incorporating resolution modeling, time of flight, normalization, attenuation, and scatter corrections. Gaussian post-reconstruction filtering (FWHM = 3 mm) was applied to all PET images. Reconstructed volumes consisted of 109 contiguous slices (2.03 mm thickness) of 128 × 128 voxels each (2.12 × 2.12 mm^2^). Due to excessive head motion that was caused by the stimulation, individual reconstructed time-frame from the same scan were registered to each other using a 3-D rigid body model. T1 anatomical MRI of each subject was also acquired on a 1.5T Magnetom scanner (Siemens) and parcellated using the Hammersmith maximum probability brain atlas (Hammers et al., 2003; Gousias et al., 2008). Time-activity curves were extracted for the caudate and for the cerebellar gray matter (without vermis) that was used as reference region (devoid of specific dopamine D2-like receptors). For additional information, the original study can be found in Fonteneau et al. (2018).

Regional TACs were submitted to modeling with lp-ntPET and b-ntPET with prior intervals set to $R_{1}\sim U\left(1,2\right)$, $k_{2}\sim U\left(0,0.5\right)$, $k_{2a}\sim U\left(0,0.1\right)$, γ~U(-0.5,0.5), $t_{D}\sim U\left(35,90\right)$, $\Delta_{t}\sim U\left(5,25\right)$, α~U(0,20). For the classic lp-ntPET approach, the basis functions were chosen by setting the extreme values for *t*_*D*_ and α accordingly, with a step of 30 s for *t*_*D*_ and *t*_*P*_, and a step of 0.5 for α.

## 4. Results

### 4.1. Simulated Dataset

In this section we present the results obtained using the 21 simulated [^11^C]raclopride dynamic PET scans. Results are shown for the caudate and accumbens regions only, which by their difference in size, exhibit two different levels of noise. Caudate as well as putamen are large regions and measured TACs are less noisy than TACs measured from the accumbens.

#### 4.1.1. Illustration of the Sampling Process

**Figure 4:**
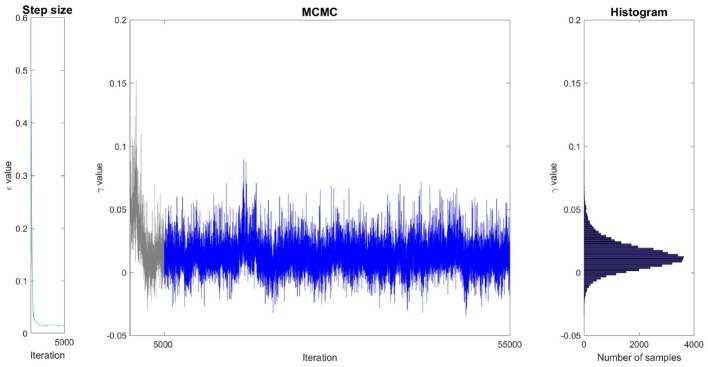
Illustration of the sampling of parameter γ on a single ROI (caudate, condition DR25). **(Left)** Evolution of the step size during the burn-in phase. **(Middle)** Generated samples (Markov Chain). The grayed region corresponds to the burn-in phase whose samples are withdrawn. **(Right)** Histogram of the remaining samples corresponding to the estimation of the marginal distribution of γ.

Figure 4 illustrates the sampling process and how it is associated with probability distributions. In this example, the Markov Chain associated to the magnitude parameter γ was initialized randomly at a high value. Consequently, the value of γ generally decreases during the first iterations to reach the ergodic distribution between −0.02 and 0.05, after around 3,000 iterations. During the first iterations, too many proposed samples have been rejected by the Metropolis-Hastings process (the acceptance rate was below $\frac{1}{2}$) so the step size was automatically lowered until it converged to a value close to 0.015 after around 1,500 iterations. After withdrawing the samples that have been drawn before convergence (during burn-in), the remaining samples are ensured to be drawn according to the marginal distribution associated to γ. Histogramming these samples gives us the shape of the distribution. One sees that in this case, ϵ converged in about 1,500 iterations, and the MCMC chain converged in about 3,000 iterations. Our choice of 5,000 iterations for burn-in is by far sufficient. It is important to keep in mind that the sampling is performed in an 8-dimensional space. Each sample of γ is associated with the seven values of the other parameters. Figure 4 only illustrates the projection of these samples in the dimension associated to γ.

#### 4.1.2. Bayesian Inference

**Figure 5:**
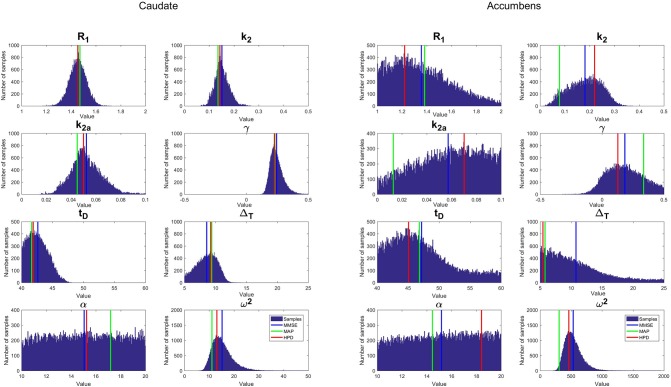
Example of the marginal distribution estimated for each parameter with b-ntPET and for a single simulated subject at condition DR25. Vertical lines show the inferred value of each parameter according to the proposed estimator HPD (red) compared to popular estimators MMSE (blue) and MAP (green). **(Left)**, caudate; **(Right)**, accumbens.

Figure 5 shows an example of marginal distribution estimated for each parameter of one subject for both caudate and accumbens (condition DR25). These marginal distributions correspond to the projection of all samples in each dimension of the parameter space. Our results showed that the distribution of most parameters were hill-shaped, which demonstrates that a single value is preferred, except for α, that exhibits a rather uniform distribution, and expresses that in this case the parameter α is non-informative. Note that the probability distributions for the accumbens exhibit larger dispersions than for caudate due to the higher noise in the accumbens TACs. That testifies that the estimations are less reliable in the case of accumbens. Such information was not available in the original version of lp-ntPET. Figure 5 also shows the difference between the different estimators. Since HPD is based on a mode-seeking algorithm, its estimations correspond to the top of the probability distributions, whereas MMSE is deviated when the probability distribution is asymmetrical. MAP, which corresponds to the single sample whose posterior probability is the highest, does not seem to belong to most probable intervals. That can be explained by the fact that the drawn samples are relatively sparse in the 8-dimensional parameter space.

#### 4.1.3. Accuracy

**Figure 6:**
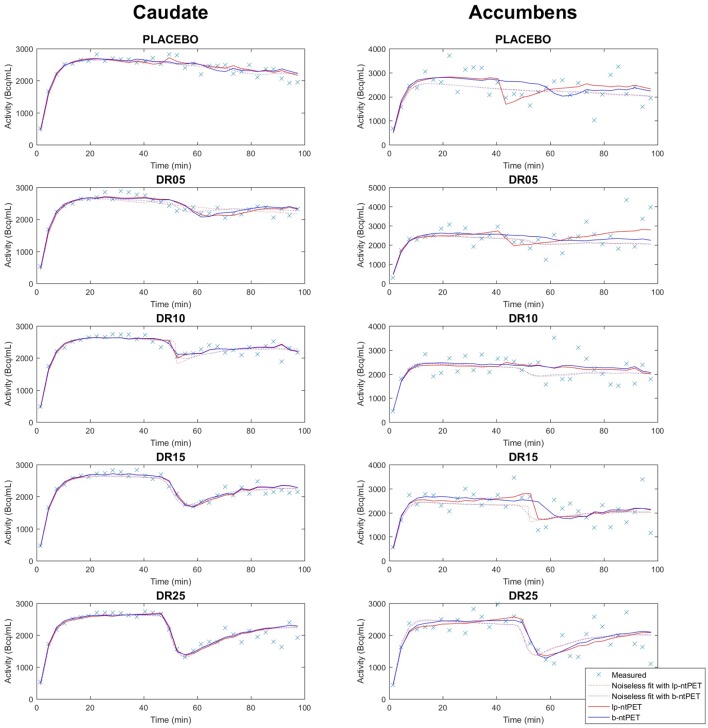
Example of simulated TACs and fitted curves obtained with lp-ntPET (red) and b-ntPET (blue). The fitted curves on the mean TACs over all subjects (here labeled *noiseless* fit) are also shown on this figure for both methods, as reference. **(Left)**, caudate; **(Right)**, accumbens.

Figure 6 shows an example of a measured TAC for each condition and how it is fitted with both methods lp-ntPET and b-ntPET. One can see that modeled TACs with b-ntPET are generally smoother than the ones modeled with lp-ntPET. On this example, b-ntPET seems to be less conducive to false detections, with lp-ntPET detecting a wider displacement in the placebo condition for accumbens.

**Figure 7:**
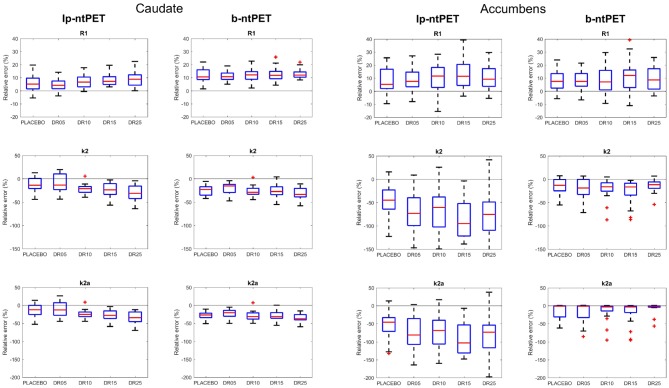
Accuracy of the estimations of the kinetic parameters (*R*_1_, *k*_2_, and *k*_2*a*_) with lp-ntPET and b-ntPET. The relative errors (in %) on the estimations are given for each condition. **(Left)**, caudate; **(Right)**, accumbens. Whiskers represent 1.5 times the IQR.

Figure 7 shows the relative errors on the estimations of the kinetic parameters for all subjects for each condition. Both methods led to kinetics estimates with similar accuracies for the caudate region. Nevertheless, with b-ntPET, the estimations of the kinetic parameters were closer to the reference for the accumbens where the noise was higher More importantly, a one-way ANOVA analysis showed that the bias on the estimated kinetic parameters *R*_1_ and *k*_2_ for the caudate region depended on the magnitude of the discharge with lp-ntPET (*p* < 0.05), whereas it remained stable across conditions with the b-ntPET estimations.

**Figure 8:**
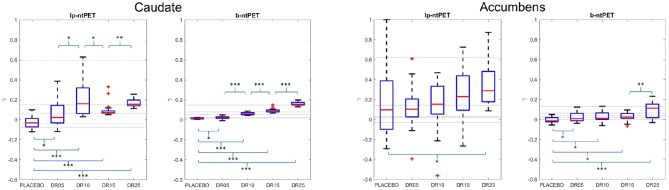
Estimates of the magnitude parameter γ across conditions obtained with lp-ntPET and b-ntPET. Horizontal lines correspond to the reference values that are obtained by fitting the *noiseless* TACs with each method (lp-ntPET and b-ntPET). **(Left)**, caudate; **(Right)**, accumbens. Whiskers represent 1.5 times the IQR. (Paired *t*-test: *p < 0.05; **p < 0.001; ***p < 0.0001).

Figure 8 shows the estimates of the magnitude parameter γ for all subjects and for all conditions. The reference value of γ was obtained by fitting the *noiseless* TACs with both methods. These reference values are represented as horizontal lines in the figure. One can observe less outliers and a lower variability in the estimations of γ with b-ntPET than with lp-ntPET. More interestingly, the sensitivity of detection was increased with b-ntPET, as it was possible to distinguish the four magnitudes of simulated dopamine release (DR05 to DR25) from the placebo condition, for the caudate as well as for the accumbens, which is not always the case with lp-ntPET. Finally, the estimated magnitude of the release is closer to zero in the placebo condition with b-ntPET than with lp-ntPET.

**Figure 9:**
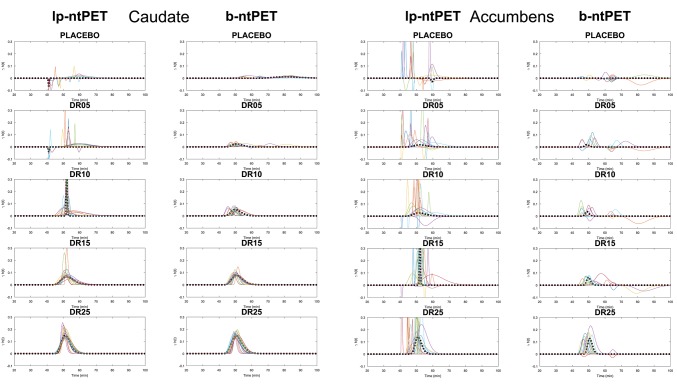
Endogeneous release curves (γ·*h*(*t*)) estimated by both methods for all conditions. Color lines represent the different subjects. The dashed line corresponds to the release curve estimated from the *noiseless* TAC by each method (reference). **(Left)**, caudate; **(Right)**, accumbens.

Figure 9 shows the endogenous release curves (γ·*h*(*t*)) that were estimated by both methods. One can observe that b-ntPET estimated released curves that were closer to the reference and with less variability. Moreover, lp-ntPET presents more aberrant curves in every condition, especially for the accumbens. Finally, lp-ntPET presents more false detections in the placebo condition.

**Figure 10:**
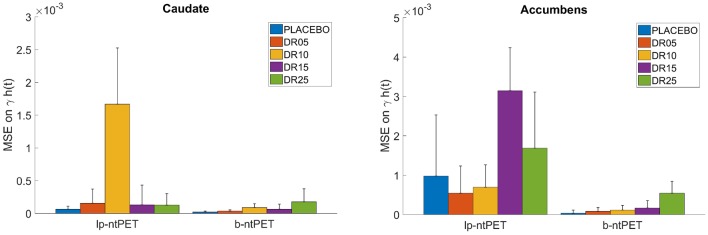
Mean Squared Error (MSE) computed between each individual estimated release curve (γ·*h*(*t*)) and the reference estimated from the *noiseless* TACs, for each estimation method and condition. **(Left)**, caudate; **(Right)**, accumbens. Error bars show the standard deviations.

Figure 10 shows the Mean Squared Error (MSE) between each individual estimated curve γ·*h*(*t*) and the references obtained from the *noiseless* TACs. B-ntPET presents a generally lower MSE than lp-ntPET, especially when high level of noise is present, such as in the accumbens. This suggests a better identification of the shape on the endogenous release. B-ntPET also presents a lower inter-subject variability.

### 4.2. Experimental Brain Cat Dataset

#### 4.2.1. Dose Effect Gamma Response

**Figure 11:**
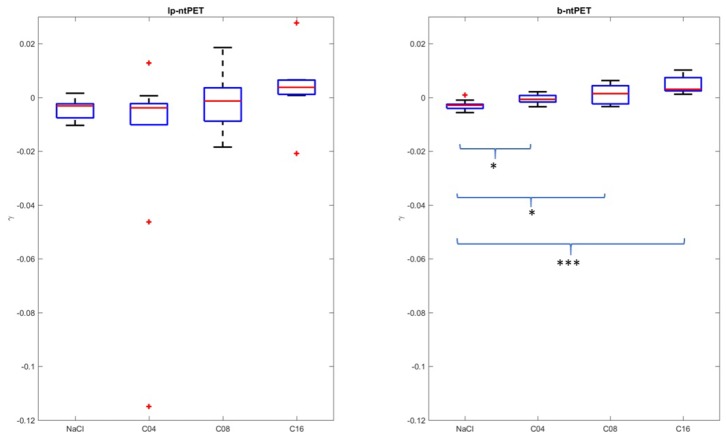
Magnitudes of the displacement (γ) estimated by both methods in the hippocampal region. The conditions NaCl, C04, C08, and C16 correspond to saline injection, and injections of 0.04, 0.08, and 0.16 mg/kg of 5-HT_1A_ agonist, respectively. Whiskers represent 1.5 times the IQR. (Two-sample *t*-test: *p < 0.05; ***p < 0.0001).

The estimations of the γ parameter obtained for the brain cat real dataset, in the hippocampus and for each individual, are reported in Figure 11. Lp-ntPET was not able to evidence any significant differences between the magnitude of the various displacements induced by pharmacological challenge compared to the control condition (NaCl injection). In contrast, differences in the magnitude of the displacement measured with b-ntPET between every dose condition and control condition (Figure 11) were significant. In addition, characterization of the gamma parameter by b-ntPET showed a lower interindividual dispersion and no outliers.

A linear correlation between the injected dose and the γ response (dose effect) could be computed with an acceptable Pearson coefficient of *r* = 0.73 (*p* < 0.0001, lower and upper bounds for 95% confidence interval = [0.52;0.85]), whereas dispersion of estimated γ parameter failed to show this correlation with lp-ntPET with a Pearson coefficient of *r* = 0.21 (*p* = 0.21, lower and upper bounds for 95% confidence interval = [−0.12;0.51]).

#### 4.2.2. Characterization of the Displacement

The estimated time-course of the radiotracer clearance from the ROI as a percentage of the baseline state $k_{2a}^{\%\text{baseline}}\left(t\right)$ defined in Equation (12) has been calculated for both methods (Normandin et al., 2012; Angelis et al., 2019).

(12) $$\begin{array}{r} k_{2a}^{\%\text{baseline}}\left(t\right)=100\cdot \frac{k_{2a}+\gamma \cdot h\left(t\right)}{k_{2a}} \end{array}$$

**Figure 12:**
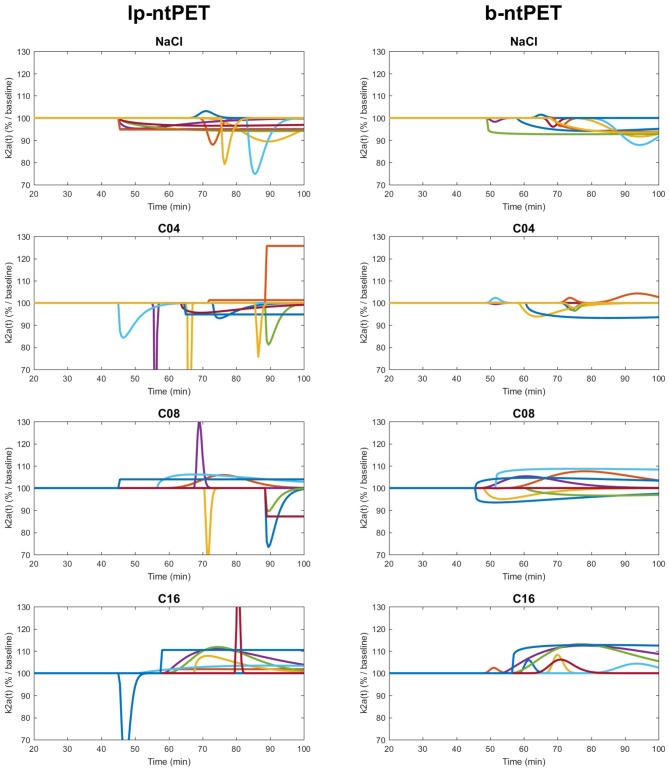
Individual $k_{2a}^{\%\text{baseline}}\left(t\right)$ curves (left and right regions for each cat), obtained with lp-ntPET **(Left)** and b-ntPET **(Right)** approaches, for each condition. The different acquisitions are represented with different colors. Both hippocampus left and right are presented.

Both lp-ntPET and b-ntPET were able to model the temporal variation of the efflux rate induced by drug competition at the individual level (Figure 12). The curves obtained with lp-ntPET presented disparate patterns and led to non-interpretable results. In contrast, the responses modeled with b-ntPET were more homogenous across subjects and the amplitude of the curves increased with the injected dose.

### 4.3. Experimental Brain Human Dataset

**Figure 13:**
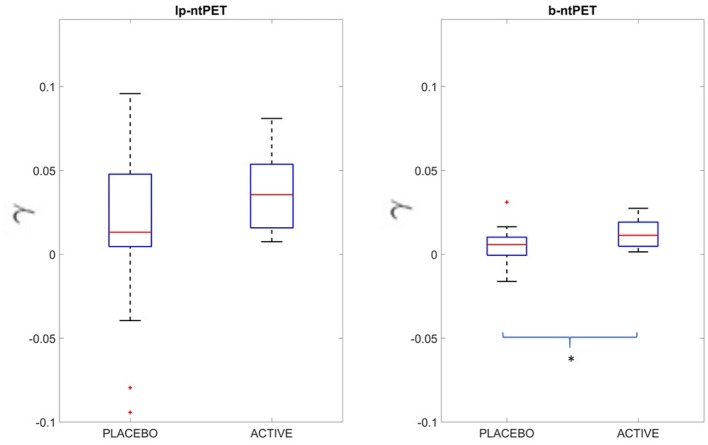
Estimated γ value in the right caudate for both methods lp-ntPET and b-ntPET. Contrary to lp-ntPET, b-ntPET allows the dissociation of the two groups (Two-sample *t*-test: *p* < 0.05).

Results from the experimental human brain raclopride PET study showed that the b-ntPET resolution method reduced uncertainties of the model parameter estimates. Figure 13 shows boxplots of the γ parameter estimated by both methods for the right caudate of all subjects. In our study, with lp-ntPET, individual γ estimates ranged from −0.04 to 0.1 with some negative outliers for the placebo group, and from zero to less than 0.1 for the active group. No statistical difference was found between the active and the placebo groups. However, the estimates produced with b-ntPET showed a much lower dispersion and consequently a significant difference was detected between groups (*p* < 0.05).

**Figure 14:**
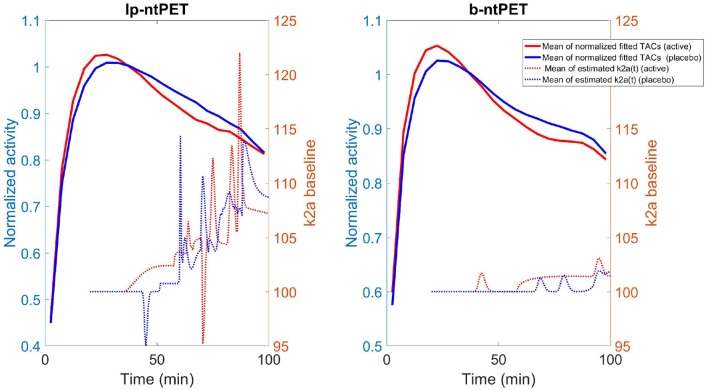
For both methods, solid line: the mean of the endogenous releases estimated for the active (red) and placebo (blues) groups using both methods; dashed line: the mean of the associated $k_{2a}^{\%\text{baseline}}\left(t\right)$ curve.

The ability of ntPET to model discharge curves is illustrated in Figure 14. Mean TACs across subjects, pooled by group (normalized by the activity of the three frames preceding the tDCS stimulation) are shown (left scale). On the right scale, mean displacement curves, per group, are plotted. With the lp-ntPET model, displacement curves were noisy and increased from the triggering of the stimuli to the end of the experiment for both the placebo and the active groups, reducing their discrimination. With the b-ntPET model, the mean discharge curve was flat for the placebo group, with the exception of small humps near 70–80 min post injection. For the active group, a hump was visible at the start of the tDCS stimulus, then it was flat before rising up at 60 min. $k_{2a}^{\%\text{baseline}}\left(t\right)$ curves were much less noisy when estimated with b-ntPET. These results are in accordance with the original publication (Fonteneau et al., 2018), where the binding ratio parameter of the right caudate was significantly different between groups in the time interval 40–55 min post tDCS.

## 5. Discussions

The possibility to characterize and quantify from a single dynamic PET experiment the system response to a stimulus or to an exogenous administration of a centrally acting cold compound provides a unique tool for the *in vivo* exploration of functional neurochemistry and psychopharmacology. With the advent of simultaneous PET/MR acquisitions, this methodology used in single PET/fMRI protocol paradigms, involving stimulation or pharmacological challenges, will open the door to the simultaneous characterization of the direct response of the neurotransmission system under investigation, as well as the mapping of the induced brain activity, with enormous potential in neurology and psychopharmacology. Several methods have been proposed to analyze neurotransmission systems under non steady state regime, such as the LSSRM and the lp-ntPET, and used for the analysis of experimental animal and human studies. Mapping of increased dopamine release induced by motor planning task was first shown in a [^11^C]raclopride human study using the LSSRM method (Alpert et al., 2003). Lataster et al. (2011) investigated the *in vivo* dopamine release in the human prefrontal cortex in response to a psychosocial stress challenge, using the radioligand [^18^F]fallypride and the same analysis method. Similarly, Ceccarini et al. (2012) detected striatal and extrastriatal reward-induced dopamine release in humans. Kim et al. (2014) revealed for the first time that different temporal patterns were involved in the dopamine response to smoking using the more advanced lp-ntPET method to analyze [^11^C]raclopride PET data of subjects smoking cigarettes during the acquisition. The same technique was used to associate the *in vivo* displacement of [^11^C]raclopride with observed behavioral changes of awake, freely moving rats following the administration of amphetamine (Angelis et al., 2019; Kyme et al., 2019).

In this work, we introduced a novel resolution method for ntPET models, called b-ntPET, that addresses some shortcomings and limitations of the current lp-ntPET model, and whereby the parameter estimation relies on a Markov-Chain Monte-Carlo (MCMC) sampling in a Bayesian framework, allowing the joint estimation of the noise level and the model parameters as well as the use of prior knowledge to guide the estimation process toward realistic solutions.

### 5.1. Performance of the Parameter Estimations

The use of well-controlled simulated PET data allowed us to show that b-ntPET produced more reliable estimates of *R*_1_, *k*_2_, and *k*_2*a*_ with higher accuracy and lower variability than lp-ntPET (Figure 7). This was especially the case in high noise context, such as for the accumbens, which is a relatively small region. These kinetic parameters rule the classical exchanges between the plasma and the free, non-specific and bound compartments as in the SRTM methods and their estimates should not be influenced by the presence of any competing ligand. However, and unlike b-ntPET, their estimated values obtained with lp-ntPET depend on the magnitude of the displacements (Figure 7), especially in the presence of noise.

More importantly, experiments on the simulated data showed that the improvement of the estimation quality when using b-ntPET was even more noticeable when estimating the magnitude of the transient effect (Figure 8). Unlike lp-ntPET, b-ntPET was able to detect and estimate the magnitude of the displacement with a high level of accuracy and precision (reduced variability). Consequently, and contrary to lp-ntPET, differences in magnitude between groups estimated by b-ntPET were found systematically statistically significant even in high noise situations. The higher performance obtained with our approach was confirmed with the experimental cat dataset where b-ntPET allowed the detection of statistical differences between the placebo group and other conditions, while no statistical differences were found when kinetics were modeled using lp-ntPET.

As a conclusion, γ estimated with b-ntPET reliably distinguished activated vs. control groups with limited sample size of subjects. In addition, γ was able to quantify the level of endogenous release as shown in Figure 8 as well as drug effect occupancy as shown by the regression results on the cat study (see section 4.2.1).

The analysis of the marginal probability density estimated for each parameter (Figure 5) revealed that the α parameter as well as and the *t*_*P*_ and *t*_*D*_ parameters in the presence of noise (accumbens region) were poorly determined. Hence, we did not specifically studied the estimation accuracy of each parameter taken independently, but the resulting γ·*h*(*t*) curves instead. With the simulated study, we were able to quantify the MSE distance between estimated curve and the reference curve (Figure 10). Results showed that whereas lp-ntPET frequently failed to characterize accurately the transient dynamic change, b-ntPET was able to produce individual curves with right magnitude and shape compared to the reference. Lp-nPET was especially deceiving in placebo condition, in noisy context, and surprisingly in the noiseless curve of the DR10 condition.

In this last case, lp-ntPET over-estimated the value for γ with a value of 0.6 (Figure 8). One can note that the estimated release curve peaked in-between two measured points (Figure 6). It is very likely that lp-ntPET, which seeks the best solution in the least squares sense, favorized this extreme solution consisting of a very brief displacement of a high amplitude between two measured points. This problem can happen even in a low noise scenario, as demonstrated with the reference curve of the DR10 condition. Shorter frames could have reduced the risk of such overfitting issue, but would have resulted in a noisier TAC. Given the low robustness to noise of the lp-ntPET method from the other hand, a compromise regarding the duration of the frames can be hard to set. This issue clearly illustrates a limitation in the lp-ntPET implementation with regard to its dependence to the time discretization. By tackling the noise more efficiently and outputting smoother TACs, b-ntPET, on the other hand, is less under the yoke of the temporal resolution.

### 5.2. B-ntPET as a Generalization of lp-ntPET

B-ntPET can be seen as an extension of the lp-ntPET method and this for several reasons. In an ideal scenario where the parameter space is totally covered by the set of basis functions, the WLS solution returned by the lp-ntPET method corresponds to the best solution in terms of goodness of fit, which is also the solution that maximizes the likelihood *p*(***Y***|**Θ**). Yet, when using b-ntPET in the special case where non-informative priors are used, as we do in this study, the posterior distribution *p*(**Θ**|***Y***) equals the likelihood *p*(***Y***|**Θ**) (up to a normalization factor). Consequently, lp-ntPET and b-ntPET seek to optimize the same objective function. Whereas lp-ntPET uses WLS to obtain directly the single set of parameters that maximizes this objective function, b-ntPET samples the parameter space to assess the objective function in its wholeness. Denser regions of samples correspond to higher probable regions in the parameter space. B-ntPET relies on all these samples to provide the best solution. The global maximum of this objective function corresponds to both the WLS solution returned by lp-ntPET and to the solution returned by b-ntPET when the MAP estimator is used. In the absence of noise, and with moderate noise, this global maximum is well defined, and both lp-ntPET and b-ntPET with the MAP estimator may provide relevant results. However, when data are noisy, the surface of the objective function is highly non-smooth and the global optimum can be translated by local optima. This can lead to overfitting issues, with the best-fit solution becoming no longer representative of the data. In this case, the HPD estimator selects a more robust solution because it relies on the seeking of the most probable cluster of samples in the parameter space rather than the single most probable sample. In other words, a set of parameters is a good candidate for b-ntPET with HPD when it leads to high fit and when small variations on these parameters also lead to good fit. Another consequence of this regularization effect when using b-ntPET with HPD is the production of smoother characterization curves (Figures 9, 12, 14), while lp-ntPET detected a lot of short releases with high amplitude.

Another conceptual difference between lp-ntPET and b-ntPET is that b-ntPET treats all the model parameters (*R*_1_, *k*_2_, *k*_2*a*_, γ, *t*_*D*_, Δ_*t*_, α) equally and estimates them at once, whereas lp-ntPET distinguishes the linear parameters (*R*_1_, *k*_2_, *k*_2*a*_, γ) and the non-linear parameters (*t*_*D*_, Δ_*t*_, α). By doing so, we believe b-ntPET reduces the dependence between the two subsets of parameters. Figure 7 illustrates that idea by showing that the estimates of the kinetic parameters (*R*_1_, *k*_2_, *k*_2*a*_) are less sensitive to the displacement with b-ntPET than with lp-ntPET. In addition, in terms of exploration of the parameter space, by relying on a discrete set of basis functions, lp-ntPET restrains the parameter space associated to the non-linear parameters *t*_*D*_, Δ_*T*_ and α to a grid, while b-ntPET explores the parameter space continuously by using MCMC sampling. An immediate consequence is the possibility for b-ntPET to assign any value to these parameters.

One key advantage of b-ntPET is its flexibility to integrate prior knowledge on the parameters. Whereas lp-ntPET allows to restrain the estimations of *t*_*D*_, Δ_*T*_, and α to plausible solutions by setting the boundaries of the basis functions accordingly, b-ntPET allows the consideration of any kind of prior distribution for *t*_*D*_, Δ_*T*_, α as well as for the other four parameters of the model.

Finally, another benefit of b-ntPET over lp-ntPET is that the noise is jointly estimated with the parameters of the model. Nevertheless, it is worth noting that the joint estimation of the noise variance can be omitted when the variance of the noise is already known with precision, for example by using some elaborate deterministic models accounting for measured counts, frame duration, deadtime etc.

### 5.3. Computational Considerations

Whereas no particular effort was made in this work to optimize the implementation of the b-ntPET method, we decided nevertheless to discuss here its computational efficiency and the existing options for improvement. As a very first observation, we can say that b-ntPET is computationally slightly more demanding than lp-ntPET. While a C implementation of lp-ntPET can analyze a TAC in less than 2 s with an i7 core, our current compiled Python implementation of b-ntPET requires about 3 s. This additional cost is not truly notable for ROI-based analysis, but could become significant for a pixel-wise implementation. The computational time required by b-ntPET is directly proportional to the amount of samples drawn. In this work, we employed 55,000 samples, a number that subsequent analyses showed to be far too high, and processing time could be significantly decreased by reducing adequately this number.

The convergence speed of the MCMC is a crucial point. We assessed the convergence properties by running 50 instances of MCMC chains over the same data from random starting values. We then measured the Potential Scale Reduction Factor (PSRF) on each parameter, which is a popular tool to measure convergence rate of MCMC chains (Brooks and Gelman, 1998). The PSRF value was below 1.02 after <100 drawn samples indicating very early convergence. Fan et al. (2016) reported that they attempted to solve the same model with MCMC but their implementation could not reach convergence within a reasonable amount of time. We believe the higher convergence efficiency of our method is a nice consequence of estimating the noise's variance jointly with the parameters of the model. Indeed, the acceptance ratio during the sampling process highly depends on the accuracy of the noise variance estimate. If overestimated, too many samples are accepted and if underestimated, too many are rejected, both cases leading to poorly-mixed samples (an ineffective exploration of the parameter space) and a risk for the MCMC chain to be stuck in a local optimum. Sampling the noise variance with direct sampling instead of an acceptance-rejection scheme also improves the mixing behavior.

Finally, our current implementation can be reduced significantly by using GPU programming.

### 5.4. Perspectives on Bayesian-ntPET

**Figure 15:**
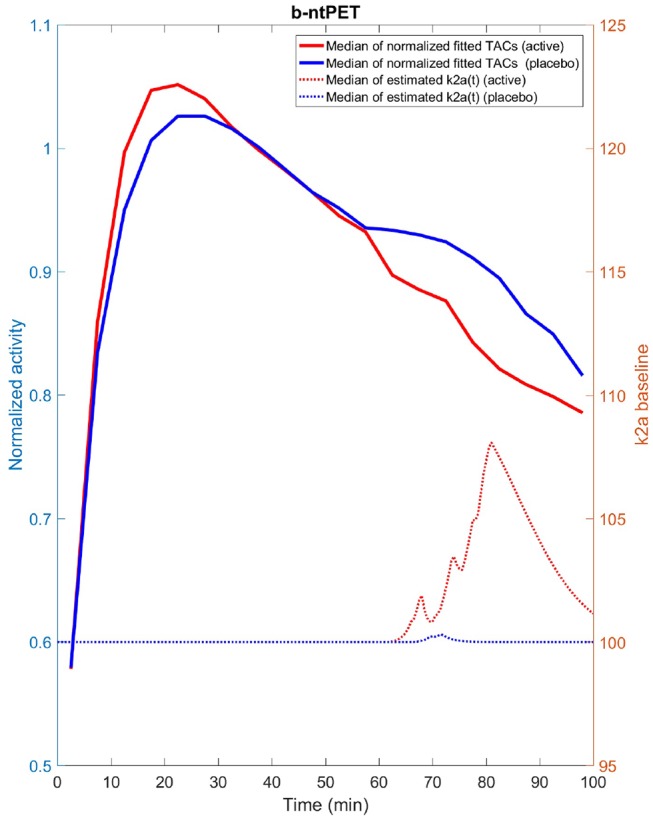
Median estimated TAC fit (solid line) as well as median estimated endogenous releases (dash line) computed for the placebo (blue) and active (red) groups from the experimental [^11^C]Raclopride study and using the multivariate HPD in lieu of the univariate HPD. We can note that the estimated releases produced with the multivariate HPD exhibit more acceptable and expected features than when the univariate HPD was used (Figure 14 right) with a quasi null release for the placebo group and a more continuous release starting after the beginning of the stimulation for the active group.

We have identified a few axes for future investigations and developments with the primary goal to improve the b-ntPET. First, in this work we did not pay much attention to the choice of the estimator, and we decided to choose HPD over the other approaches. However, inferring independently each parameter from their respective marginal distribution using the HPD estimator (and possibly with the others) is a debatable choice and may lead to suboptimal solutions. The interval with the highest probability on the marginal distributions does not always correspond to a region with high probability in the whole parameter space. One solution is to perform multivariate HPD in the whole 8-dimensional parameter space. Some preliminary tests on the real human dataset led to some very encouraging results (Figure 15). Multivariate HPD is however time consuming and needs appropriate tuning to accommodate the sparseness of the samples in the whole parameter space (the 50,000 samples are dense on marginal distributions, but are sparse in a 8-D space).

The results presented in this article were obtained using rather non-informative priors. More sophisticated priors with realistic constraints would guide the estimation process to more suitable solutions. For example, fortuitous noisy measurements in successive frames can be interpreted as brief tracer displacements leading to false detections, and setting priors that would penalize variations that are too short in time would be appropriate. Priors can also be integrated from other modalities such as fMRI or EEG according to the design of the study.

The stochastic context of the approach is also profitable. For example, one can be interested in doing model selection to classify whether or not the tracer was significantly displaced. For this purpose, Bayesian theory proposes tools to quantify the probability of the TACs to be described by a model rather than one other. Furthermore, the estimation of the posterior probability distributions provided by the methods can be exploited in a number of ways. First, confidence intervals or, more generally, information about the degree of trust on the estimation results can be easily furnished. It would also be conceivable to return more than one solution by seeking the most probable modes in the probability distribution.

In a voxel-wise implementation, spatial regularity can be enforced by a 3-D Markov Random Field, possibly supported by spatial priors such as anatomical information from MRI or CT.

In this work, the validations and the experimental applications of the method were carried out first with the [^11^C]raclopride tracer, that is known to be displaceable. This nice property is not systematically shared with other tracers, but is not specific to [^11^C]raclopride either. We showed in the third application, that the method is also applicable to [^18^F]MPPF, and lead to more exploitable results than with the reference method for this fluorinated tracer. The methodology presented here can be extrapolated to other displaceable tracers without much risk. However, two situations may challenge the efficiency of the resolution process. The first may occur when the dynamics of the transient effect can not be fitted by the current model by lack of flexibility, possibility leading to reduced sensitivity and inaccurate estimates of the magnitude of the phenomena. In this current work, the transient effect is modeled by a gamma function. We believe that the chance of violating this assumption is however low, as the gamma function can model a large variety of magnitudes and shapes. If this is not the case, the optimization scheme detailed in this study can be adapted to a new model by re-defining the likelihood function accordingly, and adapting the prior distributions to the new parameters. The second situation may occur when a displacement with a low magnitude is to be analyzed in low signal-to-noise ratio condition. We showed in this work that the joint estimation of the noise and the parameters lead to improved performance compared to the reference approach. However, for tracers inducing very low signal-to-noise ratios, the efficiency of the proposed method can be questioned and should be tested, if possible, with well-controlled simulated data before application to actual scans. Finally, the availability of the parameters' probability densities generated with the Markov chain sampling is a clear advantage of b-ntPET over other resolution approaches, as the sharpness of their profile reflects the capacity of a tracer to be displaced and therefore the suitability of the ntPET model. This is still to be tested and is beyond the scope of the current study. This will nevertheless constitute the subject of a future work with other dopamine tracers such as [^18^F]Fallypride or [^11^C]FLB45, or with tracers for other neurotransmission systems such as [^11^C]diprenorphine, which has often been the subject of competition studies.

Another question that may arise is that of the experimental design of a competition study. The stimulation time, the duration of the stimulation and its intensity are often questions asked in relation to the optimization of this design aiming at increasing the chances of detection and characterization of a discharge. In this area too, the b-ntPET approach could be useful for this optimization process, based on the study of the probability distributions and the confidence intervals that b-ntPET can generate.

Lastly, simultaneously acquired PET/MR data can also be exploited to investigate and to characterize the link between the temporal evolution of the drug uptake to the target receptors measured by PET imaging and the cerebral activity/hemodynamic response occurring after administration of drugs measured by BOLD imaging (Sander et al., 2013).

## 6. Conclusion

We have introduced a novel method named b-ntPET, for the resolution of a kinetic model involving a displacement of radiotracer produced by an endogenous neurotransmitter release or a pharmacological challenge. The proposed approach is formulated in a stochastic framework, and provides the probability distribution of the parameters of the model in addition to their estimated values. The method also supplies the level of noise in the data which is estimated jointly with the model parameters for increased robustness.

By evaluating the method on simulated and real datasets, we demonstrated that the proposed method has increased performance in terms of sensitivity and resistance to noise compared to the reference method lp-ntPET. These improvements lead to better results in terms of detection and characterization of the displacement.

As a conclusion, b-ntPET offers the possibility to reliably detect and characterize transient variation in receptor occupancy from a single PET scan.

## Data Availability Statement

The datasets generated for this study are available on request to the corresponding author.

## Ethics Statement

The studies involving human participants were reviewed and approved by Ethics committee (CPP SUD EST 6, AU1148; ANSM, A01405-42) and registered on ClinicalTrials.gov (NCT02402101). The patients/participants provided their written informed consent to participate in this study. The animal study was reviewed and approved by Comité d'éthique en expérimentation animale neurosciences CELYNE (C2EA-42).

## Author Contributions

ZI conceived the original idea, developed the theoretical formalism, and carried out the implementation. ZI, IM, JR, AR, and NC conceived the experiments. ZI, IM, JR, AR, and NC designed and carried out the numerical simulations. BV collected the data from the experimental cat dataset. BV and LZ designed the cat study and aided in analyzing the associated data and interpreting the results. CF collected the data from the experimental human dataset. CF, MS-C, and JB designed the human study and aided in analyzing the associated data and interpreting the results. ZI wrote the manuscript and designed the figures with significant input from IM, AR, and NC. NC supervised this project and was in charge of overall direction. All authors provided critical feedback and helped shape the research, analysis, and manuscript.

## Conflict of Interest

The authors declare that the research was conducted in the absence of any commercial or financial relationships that could be construed as a potential conflict of interest.
